# Computational phenotyping with the All of Us Research Program: identifying underrepresented people with HIV or at risk of HIV

**DOI:** 10.1093/jamiaopen/ooad071

**Published:** 2023-08-22

**Authors:** Xueying Yang, Jiajia Zhang, Ruilie Cai, Chen Liang, Bankole Olatosi, Sharon Weissman, Xiaoming Li

**Affiliations:** South Carolina SmartState Center for Healthcare Quality, Arnold School of Public Health, University of South Carolina, Columbia, SC 29208, United States; Department of Health Promotion, Education and Behavior, Arnold School of Public Health, University of South Carolina, Columbia, SC 29208, United States; South Carolina SmartState Center for Healthcare Quality, Arnold School of Public Health, University of South Carolina, Columbia, SC 29208, United States; Department of Epidemiology and Biostatistics, Arnold School of Public Health, University of South Carolina, Columbia, SC 29208, United States; South Carolina SmartState Center for Healthcare Quality, Arnold School of Public Health, University of South Carolina, Columbia, SC 29208, United States; Department of Epidemiology and Biostatistics, Arnold School of Public Health, University of South Carolina, Columbia, SC 29208, United States; South Carolina SmartState Center for Healthcare Quality, Arnold School of Public Health, University of South Carolina, Columbia, SC 29208, United States; Department of Health Services Policy and Management, Arnold School of Public Health, University of South Carolina, Columbia, SC 29208, United States; South Carolina SmartState Center for Healthcare Quality, Arnold School of Public Health, University of South Carolina, Columbia, SC 29208, United States; Department of Health Services Policy and Management, Arnold School of Public Health, University of South Carolina, Columbia, SC 29208, United States; South Carolina SmartState Center for Healthcare Quality, Arnold School of Public Health, University of South Carolina, Columbia, SC 29208, United States; Department of Internal Medicine, School of Medicine, University of South Carolina, Columbia, SC 29208, United States; South Carolina SmartState Center for Healthcare Quality, Arnold School of Public Health, University of South Carolina, Columbia, SC 29208, United States; Department of Health Promotion, Education and Behavior, Arnold School of Public Health, University of South Carolina, Columbia, SC 29208, United States

**Keywords:** HIV/AIDS, phenotyping, All of Us, underrepresented

## Abstract

**Objective:**

This study aims to identify the people living with HIV (PWH) and pre-exposure prophylaxis (PrEP) users in the All of Us (AoU) database by integrating information from both electronic health record (EHR)- and self-reported survey data.

**Methods:**

We identified PWH and PrEP users if they met the inclusion criterion by conditions, lab measurements, or medications related to HIV in EHR data or confirmed questions in the Survey data.

**Results:**

We evaluated the latest data release through July 1, 2022 in AoU. Through computational phenotyping, we identified 4575 confirmed and 3092 probable adult PWH and 564 PrEP users. PWH was most identified by a combination of medications and conditions (3324, 43.4%) and drug exposure alone (2191, 28.6%), then less commonly by survey data alone (608, 7.9%) and lab alone (81, 1.1%).

**Discussion and conclusion:**

Our methods serve as an overall framework for other researchers using AoU data for conducting HIV-related research.

## Introduction

The conversion from traditional survey to electronic health records (EHRs) by the health care systems and networks for clinical documentation is rapidly being done at medical centers and hospitals around the United States. While useful for clinical documentation, this transition to EHRs also yields an unprecedented opportunity to combine and analyze large clinical and ancillary datasets across multiple health care systems for research. With the advent and adoption of the EHR, researchers are now able to rapidly identify potential disease cases for clinical studies.^1–3^ To do this effectively and fully leverage the benefits of an electronic system, computable phenotype algorithms are needed that can search across billing data, laboratory data, and clinical documentation in order to perform case detection. These computable phenotype algorithms can be conceived in a manner to have high sensitivity and specificity for identifying individual subjects’ true disease status using methods borrowed from routine clinical care.^1–3^

The All of Us (AoU) Research Program constitutes a powerful platform to accelerate research focused on individuals in underrepresented groups, which aims to recruit more than 80% participants from groups that have been historically underrepresented in biomedical research.^4^ Specifically, this program define individuals with inadequate access to medical care, low household income, low education attainment, and racial or sexual and gender minorities as “underrepresented population.”^5^ AoU opened for enrollment in May 2018 and it encompasses both EHR data and survey data from a diverse regions across the United States.^4^ The recruitment methods and scientific rationale for AoU have been described previously.^4^

Mounting evidence indicates that racial and ethnic underrepresented groups carry a disproportionate burden of HIV. It is increasingly recognized that underrepresented groups defined by features other than race and ethnicity may also be at higher risk of HIV. Identifying people with HIV (PWH) and pre-exposure prophylaxis (PrEP) users in AoU would advance the HIV research in underrepresented population and promote research diversity in biomedical medicine. Although knowledge gained from observational cohort studies has dramatically advanced the prevention and treatment of disease, many observational cohorts lack diversity or do not provide comprehensive phenotype data. The phenotyping of PWH and PrEP users in AoU could accelerate biomedical research, improve health, and increase the generalizability of research findings in underrepresented population.

## Objective

Using the latest release of AoU data (both survey and EHR data), this study aims to develop computable phenotype algorithms for HIV case detection in the AoU database and examine its distribution among underrepresented groups defined by race, ethnicity, age (>75 years), disability (not able to carry out every day physical activities), sexual orientation and gender identity, income (annual household income <$35 000 US dollars), and education (less than a high school degree).

## Materials and methods

### Data sources

All data and analyses were conducted within a secure informatic workspace provided by the National Institute of Health that allows users to access and analyze a centralized version of the AoU data. There are 3 tiers of data access, in which Public Tier contains only aggregate data; Registered Tier contains individual-level EHR, wearables, and survey data; and Controlled Tier contains genomic data in addition to Registered Tier. We used release version number 7, which comprises data from all participants who enrolled from the beginning of the program on May 18, 2018 to June 1, 2022 at the Controlled Tier data.^4^

### Ethics

The AoU Research Program protocol has been previously published.^6^ The AoU protocol and materials have been approved by a dedicated institutional review board, the All of Us Institutional Review Board.

### Participants

As of January 31, 2023, AoU has harmonized from over 640 institutional sites contributing data for over 592 000 participants using the Observational Medical Outcomes Partnership (OMOP) Common Data Model (CDM). Among them, over 410 000 participants completed all initial steps of the program including consenting, agreeing to share EHR, and completing the first 3 surveys (ie, Basics, Overall Health, and Lifestyle).^7^ Participants’ identifiers have been removed before data were released to researchers. EHR and survey data are normalized by the OMOP CDM.

### Cohort identification

The HIV cohort is defined as individuals who have been diagnosed or laboratory confirmed HIV infection, and whose medication history indicating HIV specific treatment. To identify this cohort, we used several tables from the medical records including *condition occurrence*, *drug exposure*, and *measurement domains in EHR data from January 1, 1981 to July 1, 2022*. Specifically, in EHR data, we used HIV diagnosis code (ICD-10 codes, SNOMED Codes) in the “*condition occurrence*” table, HIV lab tests (LOINC codes) in the “*measurement*” table, and HIV drug exposure in the “*drug exposure*” table excluding PrEP. We adapted the concept code set from another EHR data platform by counseling the clinicians to finalize the list of concept ID for each domain.^8^ In the drug exposure, medications used for treating HCV were excluded. The PrEP users were defined as patients who were lab negative in measurement domain and taking emtricitabine and tenofovir disoproxil fumarate (FTC+TDF) or emtricitabine and tenofovir alafenamide (FTC+TAF) only in the “*drug exposure*.” To identify laboratory confirmed HIV cases, we first used a concept set of HIV specific laboratory tests to identify individuals who received HIV tests. In addition to considering HIV screen tests, our phenotype considered laboratory procedures commonly prescribed for patients with known HIV status (such as HIV viral load). From HIV screening results, we identified those whose laboratory results are “detected,” “HIV-1 positive,” “positive,” “reactive,” “abnormal,” “high.” For HIV viral load result, individuals with numeric values such as “26” and “1.95,” and >200 copies/mL (or log transformation equivalent) were included. To identify HIV-positive patients well controlled on ART, we also include individuals who were on ART and had at least 2 undetectable viral loads test (<200 copies/mL) as confirmed cases. In addition to medical records, we also used survey responses to identify HIV cases, which were collected from October 1, 2018 to June 30, 2022. Specifically, we used the OMOP CDM concept ID (1384391) to identify individuals who have affirmatively responded to the survey question “*Has a doctor or health care provider ever told you that you have or had any of the following Infectious diseases?*” *or* “*Are you currently prescribed medications and/or receiving treatment for HIV/AIDS?*” *i*n the “*Personal Medical History*” survey. We identified individuals who have responded “Infectious Disease Condition: HIV/AIDS.”

**Figure 1.**
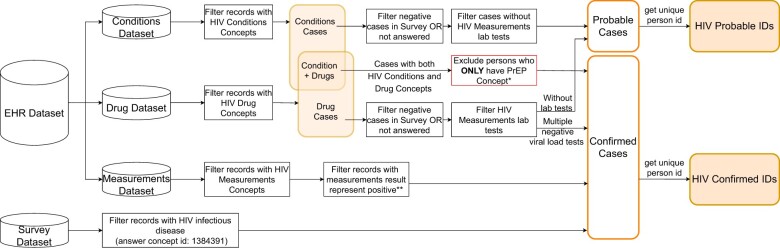
Pipeline for data extraction, data integration, and cohort construction. *PrEP user was defined as: (1) individuals with negative HIV Measurement lab test; (2) patients with either or both of following: (a) emtricitabine+tenofovir disoproxil fumarate (any dose; any type of dose); (b) emtricitabine+tenofovir alafenamide (any dose; any type of dose) Note: just one drug or more than 2 drugs are not considered as PrEP; (3) among all drug records of a patient, no other combination of drugs other than above. **We have differentiated HIV screening and viral load test in algorithm computing. For HIV screening test results, we selected the response values as “Detected,” “HIV-positive,” “Positive,” “Reactive,” “Abnormal,” and “High.” For the numeric values of viral load, we consulted the clinician on each item and included those fit to the clinical expertise. For viral load test, we conservatively used VL test value of >200 copies/mL (log(values)>log (200), log 10(values)>log 10(200)) as the threshold to be indicative of HIV infection. To identify HIV-positive patients well controlled on ART, we also include individuals who were on ART and had at least 2 undetectable viral loads test (<200 copies/mL) as confirmed cases.

After consulting an HIV clinician, PWH were categorized into confirmed and probable HIV cases. Confirmed PWH were defined as: (1) positive response to survey question; (2) individuals with a confirmed lab result; and (3) individuals with a positive response in both condition and drug exposure domain (excluding those only taking PrEP medications). Individuals with positive response in “condition” domain but with missing information in lab results and negative response/missing information in Survey dataset were defined as probably PWH. Figure 1 shows the logic flow for cohort construction. HIV relevant medical records and survey responses were linked back to *patient* table where patient demographics data were obtained, and duplicated patient IDs were consolidated.

## Results

**Figure 2.**
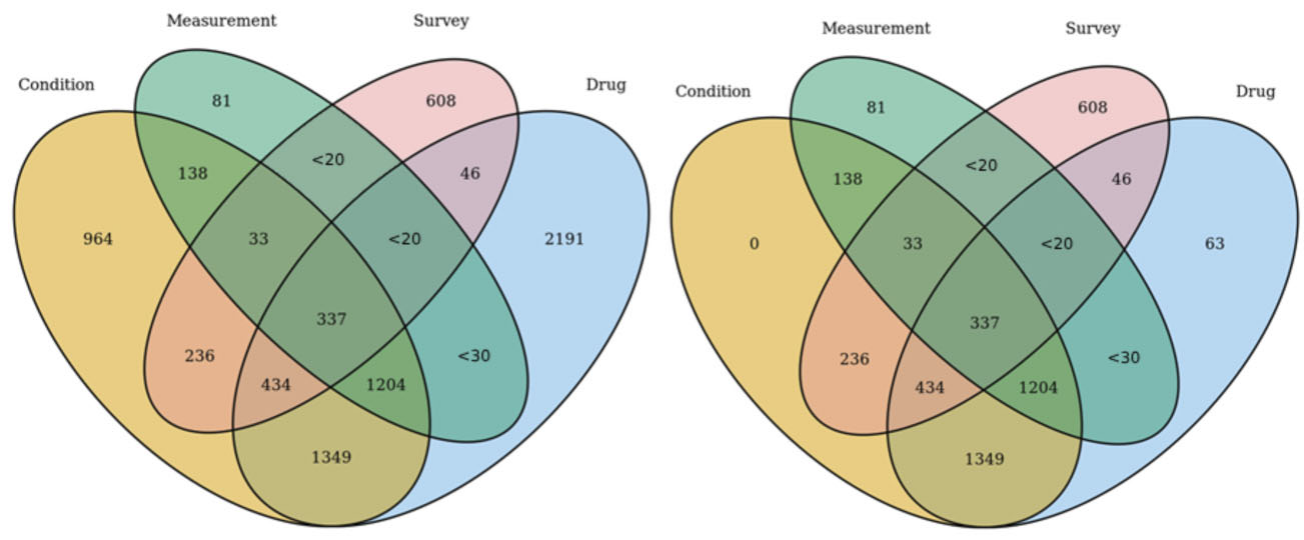
Venn diagram displaying the counts of patients identified as the total (left) and confirmed (right) cases based on conditions, labs, medications, and survey in All of Us. *Note: <20: Per All of Us policy, cell size less than 20 were masked for patients’ confidentiality concerns.*

The latest AoU data release was through July 1, 2022. Using various domain tables in EHR data and survey data, we identified 7667 adult PWH, including 4575 confirmed and 3092 probable cases. Among all the confirmed and probable cases, we identified 4695 PWH using condition table, 5602 PWH using drug exposure table, 1839 PWH using lab measurement table, and 1714 PWH using survey question. Among the confirmed cases, we identified 3731 PWH using condition table, 3474 PWH using drug exposure table, 1839 PWH using lab measurement table, and 1714 PWH using survey question. Among the probable cases, 964 and 2128 were identified by condition and drug exposure, respectively. No cases were identified by measurement and survey in the probable cases. We also identified 569 PrEP users using drug exposure table and condition table (lab confirmed negative). PWH were most commonly identified by a combination of medications and conditions (3324, 43.4%) and drug exposure alone (2191, 28.6%), then less commonly by survey data alone (608, 7.9%) and lab alone (81, 1.1%) (Figure 2).

Among the confirmed and probable cases, 71.1% aged 50+years, 41.0% were Black or African American, 15.2% were Hispanic or Latino, 40.1% were never married, 92.0% attained high school degrees or above, 12.5% has physical disability, 49.9% had income <$35 000 US dollars, and 90.8% had health insurance. Most of the social demographic distributions between the confirmed and probable cases were significantly different. For example, individuals who are males (66.5% vs 51.4%), Blacks or African American (49.2% vs 28.8%), Hispanic or Latino (17.5% vs 11.7%), Gay (38.1% vs 15.7%), and low income (<35 000 US dollars: 59.2% vs 36.1%) accounted for a larger proportion among confirmed cases than probable cases. For the potential PrEP users, most of them were male (72.4%), White (51.1%), non-Hispanic or Latino (74.0%), gay (50.8%), have a high education level (high school degree or above: 95.4%), and have health insurance (90.5%) (Table 1).

**Table 1.** Socio-demographic distribution of the identified PWH and PrEP users.

Characteristic		PWH			PrEP
	Total *N* (%)	Confirmed *N* (%)	Probable *N* (%)	*P*-value	*N* (%)
*N* (%)	7667	4575	3092		564
Gender				<.001	
Female	2728 (35.6)	1348 (29.5)	1380 (44.6)		115 (20.4)
Male	4632 (60.5)	3042 (66.5)	1590 (51.4)		409 (72.5)
Not man only, not woman only, prefer not to answer, or skipped	307 (4.0)	185 (4.0)	122 (4.0)		40 (7.1)
Age (mean, SD)	56.11 (14.0)	54.58 (12.4)	58.36 (15.7)	<.001	44.26 (13.8)
Age group (years)				<.001	
18-29	318 (4.2)	152 (3.3)	166 (5.4)		82 (14.5)
30-39	883 (11.5)	545 (11.9)	338 (10.9)		177 (31.4)
40-49	1017 (13.3)	687 (15.0)	330 (10.7)		114 (20.2)
50-64	3377 (44.1)	2299 (50.3)	1078 (34.9)		140 (24.8)
65+	2072 (27.0)	892 (19.5)	1180 (38.2)		51 (9.0)
Race				<.001	
Asian/other/unknown^a^	1578 (20.6)	1029 (22.5)	549 (17.8)		162 (28.7)
Black or African American	3142 (41.0)	2252 (49.2)	890 (28.8)		114 (20.2)
White	2947 (38.4)	1294 (28.3)	1653 (53.5)		288 (51.1)
Ethnicity				<.001	
Hispanic or Latino	1163 (15.2)	801 (17.5)	362 (11.7)		124 (22.0)
Not Hispanic or Latino	6176 (80.6)	3566 (78.0)	2610 (84.4)		417 (73.9)
Unknown^b^	328 (4.3)	208 (4.6)	120 (3.9)		23 (4.1)
Marital status				<.001	
Divorced/separated/widowed	1761 (23.0)	1084 (23.7)	677 (21.9)		77 (13.7)
Married/living with partner	2468 (32.2)	1129 (24.7)	1339 (43.3)		150 (26.6)
Never married	3075 (40.1)	2127 (46.5)	948 (30.7)		311 (55.1)
Unknown^c^	363 (4.7)	235 (5.1)	128 (4.1)		26 (4.6)
Sex orientation				<.001	
Prefer not to answer or skipped	350 (4.6)	239 (5.2)	111 (3.6)		—
Bisexual	551 (7.2)	379 (8.3)	172 (5.6)		58 (10.3)
Gay	2226 (29.0)	1741 (38.1)	485 (15.7)		288 (51.1)
Lesbian	53 (0.7)	31 (0.7)	22 (0.7)		<20
None	179 (2.3)	105 (2.3)	74 (2.4)		29 (5.1)
Straight	4308 (56.2)	2080 (45.5)	2228 (72.1)		162 (28.7)
Education				.002	
High school degree or more	7054 (92.0)	4168 (91.1)	2886 (93.3)		538 (95.4)
Less than a high school degree	267 (3.5)	181 (4.0)	86 (2.8)		<20
Unknown^c^	346 (4.5)	226 (4.9)	120 (3.9)		<20
Income				<.001	
Greater than 35 000 US dollars	2318 (30.2)	955 (20.9)	1363 (44.1)		276 (48.9)
Less than 35 000 US dollars	3823 (49.9)	2706 (59.2)	1117 (36.1)		213 (37.8)
Unknown	1526 (19.9)	914 (20.0)	612 (19.8)		75 (13.3)
Insurance				<.001	
No	412 (5.4)	278 (6.1)	134 (4.3)		32 (5.7)
Yes	6964 (90.8)	4104 (89.7)	2860 (92.5)		510 (90.4)
Unknown^d^	291 (3.8)	193 (4.2)	98 (3.2)		22 (3.9)
Medicaid (Yes)	3068 (40.0)	2188 (47.8)	880 (28.5)	<.001	133 (23.6)
Medicare (Yes)	2012 (26.2)	1083 (23.7)	929 (30.5)	<.001	47 (8.3)
Employer Or Union (Yes)	1515 (19.8)	617 (13.5)	898 (29.4)	<.001	202 (35.8)
Other health plans (Yes)	912 (11.9)	501 (11.0)	411 (13.3)	.002	84 (14.9)
Physical disability status (Yes)	960 (12.5)	593 (13.0)	367 (11.8)	.167	65 (11.5)
Number of chronic conditions					
0	4737 (61.8)	2868 (62.7)	1869 (60.5)	<.001	405 (71.8)
1	1621 (21.1)	899 (19.7)	722 (23.4)		104 (18.4)
≥2	1309 (17.1)	808 (17.7)	501 (16.2)		55 (9.8)

a Including Asian, None Indicated, More than one population, Skip, I prefer not to answer, None of these, Another single population.

b Including Skip, Prefer Not To answer; Don’t Know; None Of These.

c Including Prefer Not To Answer, Skip.

d Prefer Not To Answer, Skip, Don’t know. <20: Per All of Us policy, cell size less than 20 was masked for patients’ confidentiality concerns.

## Discussion

Using computing phenotyping, we have identified a large number of PWH and PrEP users using data from the AoU research platform. Our computing phenotyping algorithm was developed based on the strengths of previously validated algorithms,^9^ and expanded to incorporate information from survey data. Combining both EHR and survey data allows us to identify subjects that would have been missed by either algorithm alone and would promote the performance of the case identification. Comparing with prior studies, however, we did not include the number of healthcare visits as one of the criteria for phenotyping and also the algorithm was not validated against clinical notes.^10^^,^^11^

EHR-based computed phenotypes allow for the rapid identification of patients and have the added benefit of being adaptable to different healthcare systems, allowing for the development of multicenter cohorts of patients for research, clinical care, and public health initiatives. We anticipate that the individual data components of our algorithm should be readily accessible to other researchers who are using data in the AoU research platform. When comparing the sociodemographic distribution between the confirmed and probable cases, the relatively higher distribution of PWH in certain categories (eg, higher percentage of Blacks, Hispanics, male) in the confirmed group mirrors the HIV epidemic profile in the United States and revealed the representativeness of this data source for HIV epidemic in underrepresented populations. Applying our algorithms sequentially, there might be several issues that contribute to the misclassification of HIV infection status. The most common circumstance leading to mischaracterization of individual as HIV positive was incomplete metadata, where only conditions were present in the data but no record of medication or lab results. Another reason is that when relying on medications record to identify HIV cases, if one particular regimen is used to treat HBV or post-exposure prophylaxis (PEP), it can falsely categorize patients as HIV positive. Further validation studies are needed to confirm the HIV status of these probable cases.

While this cohort is specific to HIV research, our methods serve as an overall framework for other researchers to consider transferring for other patient populations or phenotyping efforts. However, our work also has several limitations. First, there might be false negative results if an individual’s diagnosis was obtained outside of the included institutions and listed solely in the narrative notes. This data could only be identified by manual extraction of data from the records, which is not feasible for this study. Second, observational studies can be subject to “volunteer bias,” as healthy people are more likely to enroll in these studies. Although recall bias could also be present, the combined use of EHR and self-reported data to ascertain outcomes and risk factors limits the impact of this type of bias. Third, we might misclassify some HBV patients or PEP users as PrEP users as some medicine to treat HIV infection (eg, Emtricitabine) can also be used to treat HBV or for PEP purpose. However, we used ICD codes (ie, B180, B181, B191) to identify the potential HBV patients among the identified PrEP users and found that none of them has corresponding HBV codes. Thus, we think such bias is not substantial. Lastly, due to the data accessibility, the validation of the algorithms based on unstructured data (eg, clinical notes) through chart review cannot be further conducted. However, the algorithm was iteratively refined through several rounds of revisions based on findings and expert feedback. We also performed a clinician annotation for a small subsample of patients. Nevertheless, we recommend future HIV researchers to use confirmed HIV cases for their analysis to ensure the accuracy of the case detection.

## Conclusion

In summary, this is the first study developing HIV case-detection algorithms based on AoU data, which represent a critical step for further evaluating the burden of HIV in underrepresented groups. This algorithm not only allows for rapid case detection of HIV individuals for researchers in AoU research platform, but also, this methodology can be extrapolated to other health care systems. We offer important evidence of the resource’s potential and report a higher burden of HIV in underrepresented groups defined by factors other than race and ethnicity. Our results underscore the urgency of addressing HIV health disparities across broadly defined underrepresented groups and point to AoU as a promising resource to advance research (eg, social determinants of health [neighborhood environment], health equity) in this area.

## Data Availability

Access to the Researcher Workbench and data is free. All researchers must be authorized and approved via a 3-step process that includes registration, completion of ethics training, and attestation to a data use agreement.
